# Blunted superior temporal gyrus activity to negative emotional expression after mindfulness-based cognitive therapy for late-life depression

**DOI:** 10.3389/fnagi.2022.1001447

**Published:** 2022-10-18

**Authors:** Weijian Liu, Hui Li, Xiao Lin, Peng Li, Ximei Zhu, Sizhen Su, Jie Shi, Lin Lu, Jiahui Deng, Xinyu Sun

**Affiliations:** ^1^Peking University Sixth Hospital, Peking University Institute of Mental Health, NHC Key Laboratory of Mental Health (Peking University), National Clinical Research Center for Mental Disorders (Peking University Sixth Hospital), Beijing, China; ^2^National Institute on Drug Dependence and Beijing Key Laboratory of Drug Dependence, Peking University, Beijing, China; ^3^Peking-Tsinghua Center for Life Sciences and PKU-IDG/McGovern Institute for Brain Research, Peking University, Beijing, China

**Keywords:** mindfulness-based cognitive therapy (MBCT), facial emotion recognition, superior temporal gyrus (STG), late-life depression (LLD), neural mechanism

## Abstract

Facial emotion recognition plays an important role in social functioning. Patients with late-life depression (LLD) often have abnormal facial emotion recognition. Mindfulness-based cognitive therapy (MBCT) is beneficial in treating depression. This study examined whether MBCT can act as an effective augmentation of antidepressants and improve facial emotion recognition in patients with LLD and its underlying neural mechanism. Patients with LLD were randomized into two groups (*n* = 30 per group). The MBCT group received an eight-week MBCT in conjunction with stable medication treatment. The other group was treated as usual (TAU group) with stable medication treatment. The positive affect (PA) scale, negative affect (NA) scale, and facial emotion recognition task with an fMRI scan were performed before and after the trial. After eight weeks of treatment, the repeated ANOVA showed that the PA score in the MBCT group significantly increased [*F*_(1,54)_ = 13.31, *p* = 0.001], but did not change significantly [*F*_(1,54)_ = 0.58, *p* = 0.449] in the TAU group. The NA scores decreased significantly in both the MBCT group [*F*_(1,54)_ = 19.01, *p* < 0.001] and the TAU group [*F*_(1,54)_ = 16.16, *p* < 0.001]. Patients showed an increase in recognition accuracy and speed of angry and sad faces after 8 weeks of MBCT. No improvement was detected in the TAU group after treatment. A significant interaction effect was found in the change of activation of the left superior temporal gyrus (L-STG) to negative emotional expression between time and groups. Furthermore, a decrease in activation of L-STG to negative emotional expression was positively correlated with the increase in PA score. The MBCT is beneficial for improving affect status and facial emotion recognition in patients with LLD, and the L-STG is involved in this process.

## Introduction

Late-life depression (LLD) is one of the most common mental disorders in aged individuals. The prevalence of major depressive disorder (MDD) is approximately 10% in people aged 60 and over (Blazer, 2003; Sjoberg et al., 2017). The recurrence rate and disability rate are high in LLD, which places a heavy burden on families and society (Alexopoulos, 2005). LLD is characterized by depression, anxiety, restless symptoms, a sense of somatic discomfort (Korten et al., 2014), and a deficit in maintaining social interactions (Washburn et al., 2016; Bomfim et al., 2019). Recognition of facial expressions is an essential part of social interactions. The interpretation of expressions and feelings of others is associated with the capacity to recognize facial expressions of emotions (Bomfim et al., 2019). Regulation of emotion is related to facial expression recognition, enabling individuals to use emotion adaptively (Celeghin et al., 2017). An inverse correlation was found between the severity of depressive symptoms and accuracy in the perception of neutral expression (Mah and Pollock, 2010). Moreover, a previous study showed that the accuracy of the recognition of angry and fearful faces decreased in elderly individuals with depressive symptoms (Orgeta, 2014). If the function of emotion recognition is damaged, the social function of depressed patients will be significantly affected, which is likely to aggravate depression and social isolation, resulting in a vicious circle. In conclusion, facial emotion recognition plays an essential role in LLD. However, it is often neglected in antidepressant therapy.

Treating LLD has always been an open question. Drug therapy has been the principal treatment measure for LLD (Flint, 1998; Chang et al., 2018). Nevertheless, traditional antidepressants have insufficient efficacy in nearly 60% of older adults due to their complex somatic situations and susceptibility to adverse events (Alexopoulos et al., 2002; Kessler et al., 2010; Nyer et al., 2013). Furthermore, evidence indicates that pure medication therapy does not modify the cognitive pattern. Thus, a non-pharmacological intervention named mindfulness-based cognitive therapy (MBCT) based on meditation and cognitive therapy is of interest (Ionson et al., 2019; Torres-Platas et al., 2019). MBCT was proven effective for depression and could improve the response rate in pharmacotherapy (Eisendrath et al., 2016). Furthermore, a randomized controlled trial showed that affective processes in remitted depressed patients improved after mindfulness training (Timm et al., 2018). Although the MBCT has been proven effective in depressive symptoms, few studies have focused on the efficacy of the MBCT for emotion recognition in depressed aged.

As MBCT, a non-drug therapy has attracted much attention in treating depression, its neuroimaging mechanism is also a subject of interest. The anterior cingulate cortex, multiple prefrontal regions, medial prefrontal cortex, striatum, and amygdala are the most frequently reported brain regions involved in the mindfulness meditation (Tang et al., 2015). Several studies also consider the temporal lobe as a critical brain region associated with mindfulness meditation, including the left inferior temporal gyrus (Zeidan et al., 2011), left middle temporal gyrus (Holzel et al., 2011), and left inferior temporal lobe (Pickut et al., 2013). However, to date, the findings regarding the neuro-mechanism of regulating facial emotion recognition with MBCT in LLD patients have been inconsistent.

Thus, we conducted an 8-week MBCT on LLD patients in the present study to investigate the change in facial emotion recognition and the related neuroimaging mechanism.

## Materials and methods

### Study design

This study was registered at http://www.chictr.org.cn (registration No. ChiCTR1800017725) and was undertaken from June 1, 2018 to February 1, 2020. Participants were randomized into the MBCT and the treatment as usual (TAU) group. The MBCT group accepted MBCT based on medication therapy for eight weeks. The TAU group merely accepted pure medication treatment for 8 weeks. All patients were asked to maintain stable medication intake throughout the trial. Clinical scales and facial emotion recognition tasks in the MRI scanner were conducted to measure the severity of symptoms and brain activity changes regarding facial emotion recognition in LLD patients before and after the treatment. The execution of the trial was approved by the Ethics Committee of the Peking University Sixth Hospital (Institute of Mental Health). The agreement number concerning human research ethics is No. 2018 (15). Informed consent was signed by all participants.

### Participants

**FIGURE 1:**
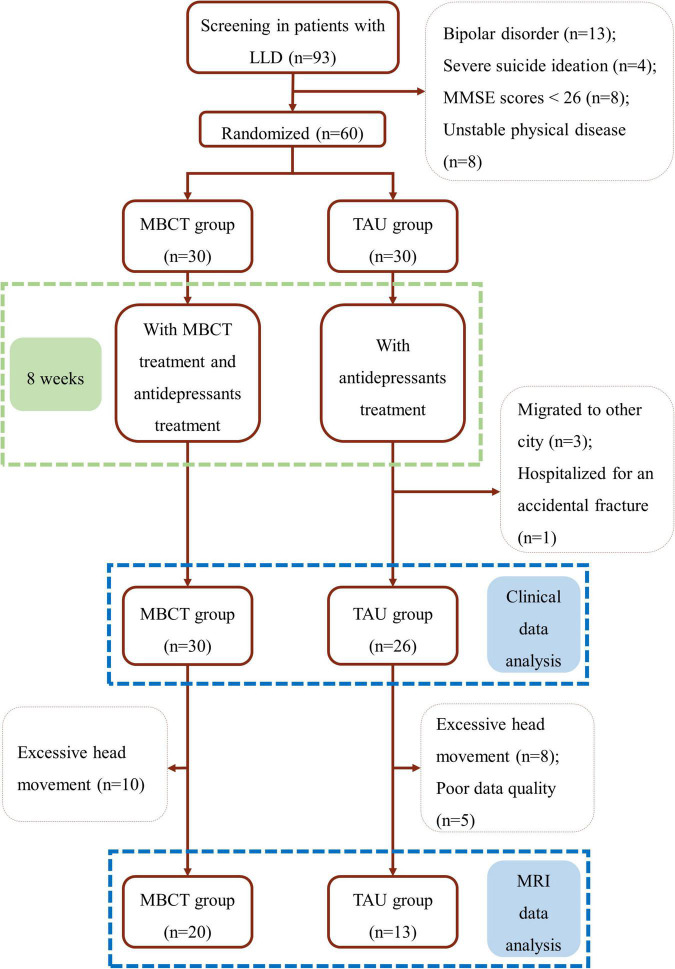
Flow chart. LLD, late-life depression; MBCT, mindfulness-based cognitive therapy; TAU, treatment as usual; MMSE, the Mini-Mental State Examination.

A sample size of 48 was calculated using GPower 3.0.10 software for a power of statistical 80% with an alpha of 5% (*p* ≤ 0.05). Considering the correction for a dropout rate of one in five (20.0%), six additional participants per group should be enrolled, resulting in a total sample size of 60 subjects (30 subjects per group). During the trial, four patients in the TAU group were lost in follow-ups. Clinical data were analyzed in 56 patients (30 in the MBCT group and 26 in the TAU group). Moreover, ten MRI data were excluded in the MBCT group and 13 fMRI data were excluded in the TAU group because of excessive head movement or poor data quality. Finally, 20 patients in the MBCT group and 13 in the TAU group were included in the final fMRI analyses (details in the flow chart, Figure 1).

Patients aged over 60 years who fulfilled the diagnostic criteria for MDD (single episode or recurrent) listed in the Diagnostic and Statistical Manual of Mental Disorders-4th edition (DSM-IV) (American Psychological Association [APA], 2000) were enrolled. The Structured Clinical Interview for DSM-IV (SCID) was used to screen participants. The diagnosis of MDD was confirmed by two experienced doctors with at least five years of clinical experience. In addition, all participants had normal visual acuity, either naked or corrected, and were right-handed. All participants took medications stably for more than two weeks before enrollment. The exclusion criteria were as follows: (1) at a high risk of suicide; (2) once or currently diagnosed with other psychiatric disorders such as schizophrenia, bipolar disorder, and substance abuse; (3) a history of neurological disorders such as epilepsy, Parkinson’s disease, and dementia; (4) current unstable severe physical diseases; (5) with a Mini-Mental State Examination (MMSE) scores less than 26; (6) taking part in psychotherapy concurrently; and (7) contraindications to MRI scans.

### Intervention

The TAU group maintained stable medications without any psychotherapies for eight weeks. Regular visits were conducted once a week in the TAU group.

The MBCT group accepted additional MBCT based on stable medication therapy. The MBCT intervention consists of eight weekly face-to-face group-practice sessions of 1–1.5 h each. Furthermore, participants performed 45 min of self-practice each day. The MBCT was led by two experienced therapists trained in mindfulness, and two other psychiatrists assisted in the face-to-face group practice session. An online communication group was established through WeChat software. All subjects were required to punch in online after daily practice and report their time and feelings regarding self-practice at home. Self-practice manuals were distributed before the treatment, and the subjects had to make a paper record after each self-practice session, including but not limited to their practice time, practice items, and any problems encountered. Participants who had two or more absences from face-to-face group practice were considered lost in follow-ups. The intervention framework and content of MBCT are shown in Supplementary Table 1.

### Clinical outcomes

The positive and negative affect scale (PANAS) and its subscales [positive affect (PA) scale and negative affect (NA) scale] were performed before and after the trial to examine the affective change in LLD patients. Hamilton depression scale-17 (HAMD-17) was preformed at baseline.

### Facial emotion recognition task

Our study administered a facial emotion recognition task during fMRI scanning with E-Prime software (Psychology Software Tools, Pittsburgh, PA, USA). The facial emotion pictures are derived from the Chinese Affective Face Picture System (CAFPS) (Xu et al., 2011). Thirty pictures with happy, sad, angry, fearful, and neutral faces were randomly presented. The pictures were shown to subjects through a visual system with the magnetic resonance machine. Two faces, one male and one female, were presented for each facial emotion image. All pictures were consistent in size, brightness, and emotional intensity.

Clear instruction and a block of practice were performed outside the magnetic resonance room before the formal task to ensure that all the participants understood the content and procedures of the experiment. Subjects were asked to identify the genders of the pictures and press the reaction box button as fast as possible. Correctness was defined as the subjects selecting the right gender for the picture. The formal task could only be carried out when the correct rate reached more than 70%. The task used the implicit paradigm of facial emotion processing. Facial emotion pictures were presented as target stimuli for 2,000 ms. Then, a “+” appears in the center of the screen for 300–700 ms. The task had two sessions, each of which had 30 trials and lasted approximately 3 min (Supplementary Figure 1). A short rest time between sessions was designed to prevent the effects of fatigue on the subjects. Each subject’s average accuracy and response time in different facial emotion type trials were calculated.

### Imaging acquisition and data processing

Participants were scanned using a 3.0 Tesla General Electric scanner (GE MR750 3.0T). High-resolution T1-weighted structural images were acquired using the following parameters: field of view (FOV) = 25.6 cm^1^, flip angle = 12°. The fMRI images were acquired with the following parameters: FOV = 22.0 cm^3^, TR = 2,000 ms, TE = 30 ms, flip angle = 90°, number of slices = 43, total scans = 240).

Image data processing was based on MATLAB 2013.^2^ Statistical parametric mapping 12 (SPM12)^3^ was used to preprocess image data. The preprocessing of the images included slice timing and realignment. Then the realigned image volumes were used to construct a mean functional image volume for each subject. The T1-weighted anatomical images were coregistered to the mean functional image and normalized to the Montreal Neurological Institute (MNI) coordinate system. After normalization, functional images were resliced into 3 mm × 3 mm × 3 mm voxels and smoothed with a 6 mm Gaussian kernel.

First- and second-level analyses were performed in NeuroElf.^3^ General linear model (GLM) analyses were conducted to identify the blood oxygen level-dependent (BOLD) signal related to facial emotion types (happy, sad, angry, fearful, and neutral faces). Six standard motion parameters from realignment, onsets, and durations of events convolved with the hemodynamic response function (HRF) were included in subject-level models. A high-pass temporal filtering method (with a cutoff of 128 s) was conducted to remove low-frequency artifacts.

As only recognition of negative facial emotions (angry, fearful, and sad) significantly changed in behavioral data analyses, one contract (negative > neutral) was constructed. A two-way analysis of variance (ANOVA) was used to examine the interaction effects of the group (MBCT and TAU groups) and time (pre- and posttreatment) on neural activation (negative > neutral), with the accuracy and reaction time of five emotional faces at baseline as covariates. After a voxel-level correction at p < 0.001 and a cluster-level at p-FWE < 0.05. The cluster extent threshold was 23 voxels (smoothness: 8 mm kernel).

### Data analysis

Clinical and behavioral data were described as the means ± standard deviations (SD) or percentages (%), and data analyses were conducted using SPSS 20.0 statistical software (SPSS Inc., Chicago, IL, United States). Comparisons of differences between the MBCT group and the TAU group were performed using the independent samples *t*-test and the chi-squared test. For the clinical accuracy and response time, repeated ANOVA was performed to compare whether there were significant differences between baseline and endpoint in the two groups. Facial emotional type and time point were regarded as intragroup factors, and the group was regarded as an intergroup factor, followed by a simple effect analysis.

Activation of a significant interactive brain region was extracted (β value). Independent samples t-test was used to detect whether changes in activation of the interactive brain region were significantly different between the two groups. Partial correlation analysis was conducted to detect the relationship between the deviation of the β value and change in clinical scales in each group with the accuracy and reaction time of five facial emotions at baseline as covariates.

## Results

### Demographic and clinical features

In our study, 60 LLD patients were enrolled (30 per group). The participants in the MBCT group were 67.66 ± 5.93 years old and 67.22 ± 5.78 years old in the TAU group. Six (20.00%) males were in the MBCT group, and 8 (26.67%) males were in the TAU group. The HAMD-17 scores were 17.67 ± 6.69 and 18.56 ± 7.00 in the MBCT and TAU groups, respectively. There were no significant differences in demographic characteristics, age of onset, duration of MDD, comorbidity, or medication use between the two groups (Table 1, all *p* > 0.05). The demographic and clinical characteristics of the MRI analysis are shown in Supplementary Table 2, and no significant differences were found.

**TABLE 1:** Demographic and clinical characteristics of the MBCT and TAU groups at baseline.

Participants	MBCT (*n* = 30)	TAU (*n* = 30)	t/χ^2^	*P*
	Mean ± SD/*n* (%)	Mean ± SD/*n* (%)		
Age (years)	67.66 ± 5.93	67.22 ± 5.78	0.29	0.776
Gender (male)	6 (20.00%)	8 (26.67%)	0.37	0.761
Education (years)	13.73 ± 2.66	12.5 ± 3.08	1.66	0.103
BMI (kg/m^2^)	22.64 ± 2.42	23.63 ± 1.83	−1.77	0.082
Married	25 (83.33%)	25 (83.33%)	0.00	1.000
Duration of Illness (months)	48.23 ± 43.36	50.23 ± 35.41	−0.20	0.846
Age of onset (years)	62.33 ± 7.26	61.96 ± 5.56	0.22	0.827
**Comorbidity**				
Hypertension	11 (36.67%)	10 (33.33%)	0.07	0.787
Diabetes mellitus	1 (3.33%)	2 (6.67%)	0.35	0.554
HAMD-17	17.67 ± 6.69	18.56 ± 7.00	−0.50	0.617
PA score	24.23 ± 6.94	23.27 ± 6.19	0.57	0.571
NA score	20.43 ± 6.24	22.53 ± 6.46	−1.28	0.205
**Type of antidepressant**				
SSRI	22 (73.33%)	23 (76.67%)	0.09	0.766
SNRI	8 (26.67%)	7 (23.33%)	0.09	0.766

BMI, body mass index; PA, positive affect; NA, negative affect; MBCT, mindfulness-based cognitive therapy; TAU, treatment as usual; SSRI, selective serotonin reuptake inhibitor; SNRI, selective noradrenalin reuptake inhibitors; HAMD-17, hamilton depression scale-17.

### Mindfulness-based cognitive therapy improved the positive affect score in patients with late-life depression

**FIGURE 2:**
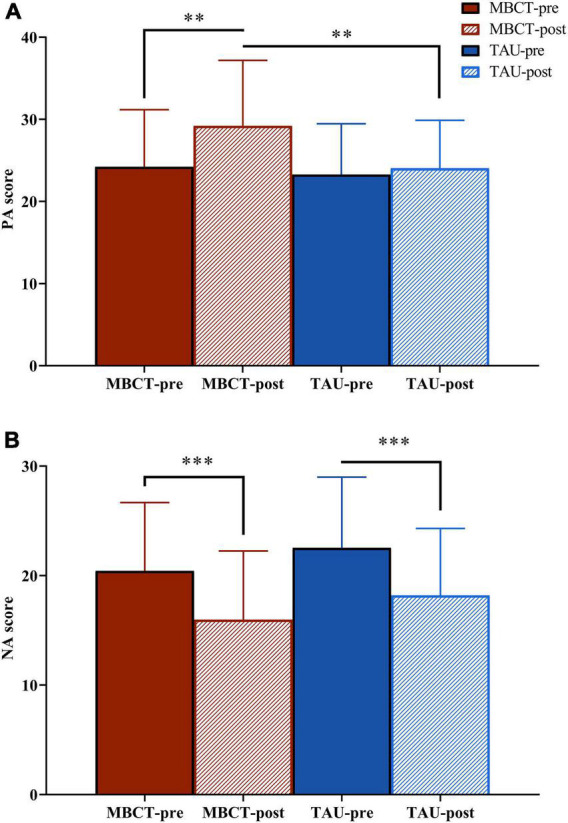
The PA and NA scores of the two groups before and after the intervention. Repeated analysis of variance. **(A)** PA score; **(B)** NA score. The red bar is the MBCT group (*n* = 30) and the blue bar is the TAU group (*n* = 26). PA, positive affect; NA, negative affect; MBCT, mindfulness-based cognitive therapy; TAU, treatment as usual. ^**^p < 0.01; ^***^p < 0.001.

All participants in the MBCT group finished the study. In contrast, four participants were lost in follow-ups with the TAU group (three participants traveled to other cities, and one was hospitalized for an accidental fracture). Finally, 30 participants in the MBCT group and 26 in the TAU group were included in the clinical outcomes and behavioral data analyses. The HAMD-17 scores decreased significantly in the two groups mentioned in our previous study (Li et al., 2022). Moreover, the results of PA and NA are shown in Figures 2A,B.

For PA, the repeated ANOVA indicated a significant time effect [*F*_(1,54)_ = 9.26, *p* = 0.004]. The effect of time × group was on the threshold of significance [*F*_(1,54)_ = 3.71, *p* = 0.059]. After treatment, the PA score in the MBCT group was significantly higher than that in the TAU group [*F*_(1,54)_ = 7.43, *p* = 0.009]. In the MBCT group, the PA score significantly increased [*F*_(1,54)_ = 13.31, *p* = 0.001) after treatment. In the TAU group, the PA score did not change significantly [*F*_(1,54)_ = 0.58, *p* = 0.449] after treatment.

For the NA, the repeated ANOVA showed no time × group effect [*F*_(1,54)_ = 0.01, *p* = 0.977] but a significant time effect [*F*_(1,54)_ = 34.97, *p* < 0.001]. The NA scores decreased significantly in both the MBCT group [*F*_(1,54)_ = 19.01, *p* < 0.001] and the TAU group [*F*_(1,54)_ = 16.16, *p* < 0.001].

### Mindfulness-based cognitive therapy improved the recognition accuracy and response time of negative facial emotions

**FIGURE 3:**
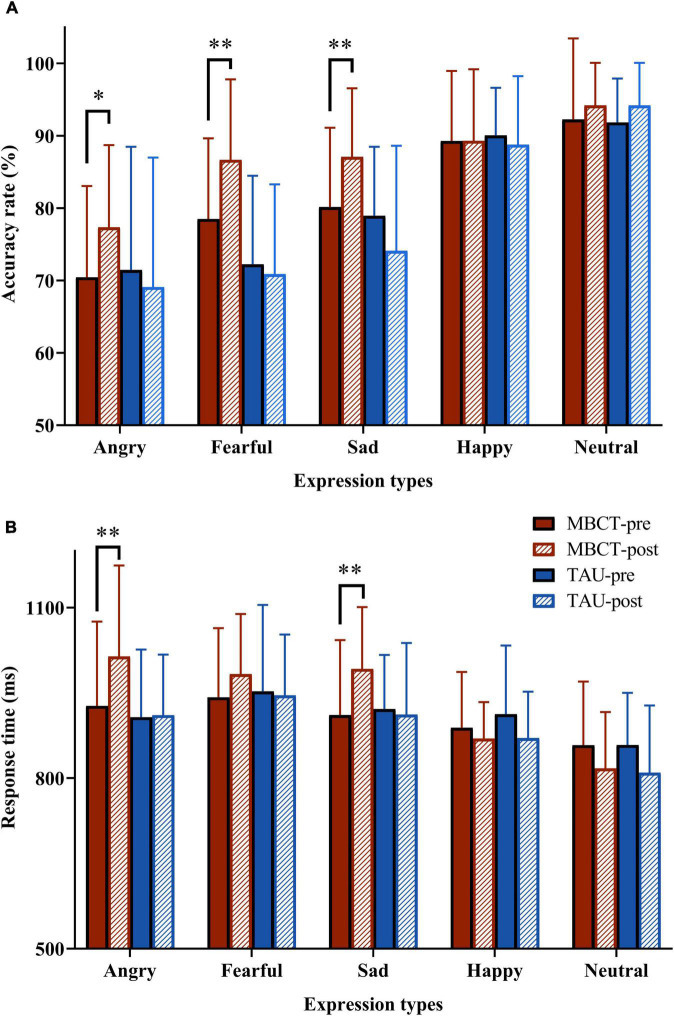
The accuracy and response time to facial emotion pictures in the two groups before and after the intervention. **(A)** PA score; **(B)** NA score. Repeated analysis of variance. The red bar is the MBCT group (*n* = 30) and the blue bar is the TAU group (*n* = 26). MBCT, mindfulness-based cognitive therapy; TAU, treatment as usual. *p < 0.05, ^**^p < 0.01, compared with MBCT-pre group.

For the accuracy of facial emotion, the repeated ANOVA showed a significant emotion types main effect [*F*_(4,216)_ = 87.34, *p* < 0.001], a non-significant time main effect [*F*_(1,54)_ = 1.97, *p* = 0.166], a significant time × group interaction effect [*F*_(1,54)_ = 7.25, *p* = 0.009], a significant emotion types × group interaction effect [*F*_(4,216)_ = 6.63, *p* < 0.001], a non-significant emotion types × time interaction effect [*F*_(4,216)_ = 1.21, *p* = 0.306], a significant emotion types × time × group interaction effect [*F*_(4,216)_ = 4.15, *p* = 0.003] and a significant group main effect [*F*_(1,54)_ = 5.50, *p* = 0.023]. Simple effect analysis revealed that in the TAU group, the accuracy of facial emotions was not significantly different between baseline and endpoint. However, the accuracy of angry (*p* = 0.011), fearful (*p* = 0.002), and sad (*p* = 0.005) expressions in the MBCT group increased significantly after the intervention (Table 2 and Figure 3A).

**TABLE 2:** The accuracy rate of facial emotion pre- and post-treatment in the MBCT and TAU groups.

Facial emotion types	MBCT group (*n* = 30)		TAU group (*n* = 26)	
	Pre-treatment (%)	Post-treatment (%)	Pre-treatment (%)	Post-treatment (%)
**Angry**	70.44 ± 12.62	77.36 ± 11.35	71.43 ± 17.04	69.08 ± 17.90
**Fearful**	78.48 ± 11.16	86.67 ± 11.12	72.22 ± 12.25	70.88 ± 12.40
**Sad**	80.14 ± 10.98	87.08 ± 9.49	78.93 ± 9.54	74.10 ± 14.51
**Happy**	89.27 ± 9.69	89.30 ± 9.88	90.05 ± 6.58	88.77 ± 9.46
**Neutral**	92.25 ± 11.20	94.19 ± 5.89	91.84 ± 6.08	94.19 ± 5.89

MBCT, mindfulness-based cognitive therapy; TAU, treatment as usual.

For the response time of facial emotion, the repeated ANOVA showed a significant emotion types main effect [*F*_(4,216)_ = 27.50, *p* < 0.001], a non-significant time main effect [*F*_(1,54)_ = 0.17, *p* = 0.681], a significant time × group interaction effect [*F*_(1,54)_ = 5.18, *p* = 0.027], a non-significant emotion types × group interaction effect [*F*_(4,216)_ = 2.36, *p* = 0.054], a significant emotion types × time interaction effect [*F*_(4,216)_ = 7.36, *p* < 0.001], a non-significant emotion types × time × group interaction effect [*F*_(4,216)_ = 1.56, *p* = 0.185] and non-significant group main effect [*F*_(1,54)_ = 1.06, *p* = 0.307]. Simple effect analysis revealed that in the TAU group, the response time of facial emotions was not significantly different between baseline and endpoint. However, the response time of angry (*p* = 0.001) and sad (*p* = 0.002) expressions in the MBCT group increased significantly after the intervention. Furthermore, the response time to fearful expressions showed a marginally significant increase in the MBCT group at the end of the study (*p* = 0.056) (Table 3 and Figure 3B).

**TABLE 3:** The response time to facial emotion pre- and post-treatment in the MBCT and TAU groups.

Faci al emotion types	MBCT group (*n* = 30)		TAU group (*n* = 26)	
	Pre-treatment (ms)	Post-treatment (ms)	Pre-treatment (ms)	Pre-treatment (ms)
Angry	926.77 ± 148.69	1014.17 ± 159.80	907.19 ± 119.06	910.50 ± 106.76
Fearful	941.94 ± 121.97	982.78 ± 106.00	952.28 ± 152.42	945.61 ± 106.81
Sad	910.97 ± 131.83	991.96 ± 109.03	921.15 ± 95.65	911.78 ± 125.82
Happy	888.48 ± 98.19	869.45 ± 64.18	912.49 ± 120.81	870.36 ± 81.59
Neutral	857.91 ± 111.96	817.05 ± 99.28	858.18 ± 91.96	809.60 ± 118.26

MBCT, mindfulness-based cognitive therapy; TAU, treatment as usual.

### Mindfulness-based cognitive therapy reduced left superior temporal gyrus response to negative emotion

As we discovered the behavioral indicators of negative facial emotions changed significantly after MBCT, the angry, fearful, and sad conditions were combined as negative facial emotion conditions.

**FIGURE 4:**
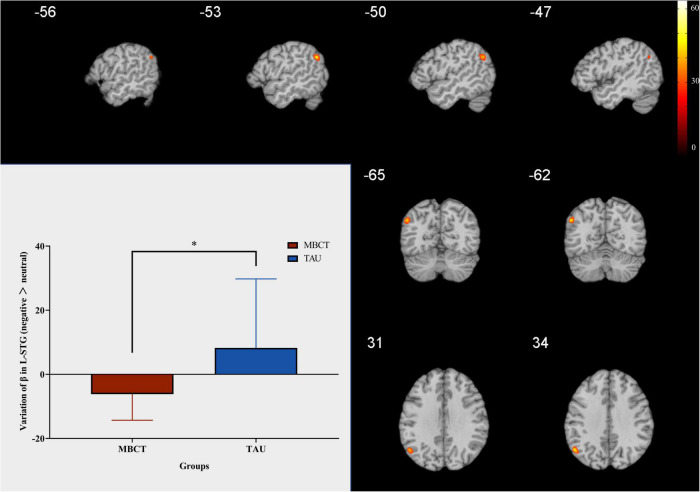
Change in L-STG response to negative facial emotion. Independent samples *t*-test. The red bar is the MBCT group (*n* = 20) and the blue bar is the TAU group (*n* = 13). The region marked in the figure is the brain region with significant interaction between the time point and the group. L-STG = left superior temporal gyrus; MBCT, mindfulness-based cognitive therapy; TAU, treatment as usual. *p < 0.05.

In the whole-brain analysis, the interaction between the time point and the group was found in only one brain region (L-STG, voxel size = 23, coordinate = −53; −62; 29). The change in activation in the L-STG in the MBCT group was significantly lower than that in the TAU group (*t* = −2,74, *p* = 0.01). The results are shown in Figure 4.

### Positive correlation between activation of the left superior temporal gyrus and the changes in the positive affect score

**FIGURE 5:**
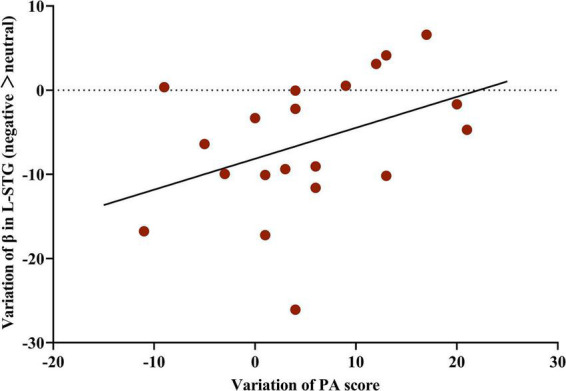
Correlation between the change in activation of the L-STG and variation in PA score in the MBCT group. Partial correlation analysis (*n* = 20) with the accuracy and reaction time of five facial emotions at baseline as covariates.L-STG, left superior temporal gyrus; MBCT, mindfulness-based cognitive therapy; PA, positive affect.

In the MBCT group, the change in activation of the L-STG was positively associated with the changes in the PA score (*r* = 0.76, *p* = 0.011, Figure 5). However, neither change in PA nor NA score was significantly associated with the change in L-STG activation in the TAU group.

## Discussion

To our knowledge, this is the first study to investigate whether MBCT would change the recognition accuracy and speed of facial emotion in LLD patients, and the underlying neuroimaging mechanism. The significant findings of this study were as follows: (1) the PA score was increased and the NA score decreased after MBCT; (2) the accuracy of angry, fearful, and sad faces were increased, and the response time of angry and sad faces increased after MBCT; (3) an interaction effect was found in the change of activation of L-STG between MBCT group and TAU group; and (4) reduction of activation of L-STG was positively associated with the increase of PA score.

In the present study, the NA score decreased in both the MBCT and TAU groups, which means that both pure medication treatment and MBCT effectively regulated emotion recognition. Nevertheless, the PA score increased only in the MBCT group but not in the TAU group. This means that MBCT showed an advantage in improving PA in LLD patients. A previous study found that reports of increased experience of positive emotions were related to MBCT (van der Velden et al., 2015). In contrast to progressive muscle relaxation, PA increased and NA decreased after four weeks of mindfulness training (Timm et al., 2018). MBCT has been shown to have emotion regulation effects in many studies, including boosting the positive mood (Hofmann et al., 2010; Goyal et al., 2014) and decreasing the negative mood (Jha et al., 2010). Consistent with prior evidence, the addition of MBCT to conventional medication therapy has an advantage in emotion regulation compared to medication alone.

In this study, the accuracy of angry, fearful, and sad faces increased, and the response time of angry and sad faces increased after MBCT. A recent meta-analysis showed that depressed patients have impaired facial emotion recognition (Dalili et al., 2015). A negative response bias toward sadness in individuals with major depression was proven by consistent evidence (Bourke et al., 2010). A previous study found that older people without depression are more accurate in recognizing anger and sad faces than depressed older people (Bomfim et al., 2019). In our study, the accuracy and response time of negative facial emotion were increased after MBCT, indicating that adding MBCT to conventional medication treatment improves facial emotion bias in elderly patients with depression. Non-judgemental acceptance and present-moment awareness during mindfulness meditation make people more sensitive to facial and emotional stimuli, which is probably the vital reason for promoting the cognitive control (Teper et al., 2013).

In neuroimaging mechanisms, we found that the reduction in activation of L-STG was positively associated with an increase in PA score. Previous research has shown that the STG plays an important role in the social cognitive functions (Allison et al., 2000; Jou et al., 2010), perception of social information (Allison et al., 2000), and emotion processing and encoding changes in facial emotion (Tseng et al., 2016). Right STG is also associated with the social anhedonia (Germine et al., 2011). In people with familial risk for psychosis, the STG relates to the facial emotion recognition (Pulkkinen et al., 2015). In children with subclinical anxiety, the STG was concerned with the facial emotional recognition (Kennedy et al., 2021). In patients with schizophrenia, the STG volume plays a role in social functions (Jung et al., 2020). In ischemic stroke patients, the ability to recognize angry expressions was only related to the STG (van den Berg et al., 2021). Moreover, after mindfulness practice, altered temporal nodal efficiency of the STG was found in aging adults (Fam et al., 2020). The above evidence supports our finding that STG plays an essential role in MBCT for patients with LLD.

Several limitations must be pointed out in this study. First, the sample size was small. Further study with a larger sample size should be performed. Second, a relative amount of MRI data was excluded after preprocessing. Given that it is not easy for older adults to remain still throughout the entire scan, it is not surprising that some of the participants were excluded for excessive head movement and poor data quality. Third, the only additional condition conducted in the control group was regular visits. A previous study regarded antidepressant medications and non-structured supportive therapy as their control condition (Ionson et al., 2019). A more convincing control condition should be performed in future studies.

## Conclusion

In conclusion, this study showed that MBCT is beneficial to older adults with LLD in improving affect and facial emotion recognition, and the L-STG plays a significant role in this process.

## Data availability statement

The raw data supporting the conclusions of this article will be made available by the authors, without undue reservation, after permission of all authors.

## Ethics statement

The studies involving human participants were reviewed and approved by Ethics Committee of the Peking University Sixth Hospital (Institute of Mental Health). The patients/participants provided their written informed consent to participate in this study.

## Author contributions

HL, LL, JD, and XS contributed to the study design. HL, XZ, SS, and XS performed the research and collected the data. WL, HL, XL, and PL performed the data analysis. WL, XL, HL, and JD drafted the manuscript. All authors reviewed the results and approved the final version of the manuscript.

## Footnotes

1
http://www.neuroelf.net

2
https://www.mathworks.com/products/matlab.html

3
https://www.fil.ion.ucl.ac.uk/spm/software/spm12/
