# Supplementary material for: Blunted superior temporal gyrus activity to negative emotional expression after mindfulness-based cognitive therapy for late-life depression
Source: Front Aging Neurosci. 2022 Oct 18;14:1001447. doi: 10.3389/fnagi.2022.1001447 (PMC9623567; doi:10.3389/fnagi.2022.1001447)
Supplement: Supplementary file 1 [file Data_Sheet_1.docx]

Supplementary Material

| **Supplementary Table 1. Intervention framework and contents of MBCT.** | |
| --- | --- |
| **Intervention framework** | |
| **The first and second sessions (breath mindfulness)** | In these sessions, the therapist introduced the pathogenesis of LLD, and explained the concept and principle of MBCT, to make the participants realize the effect of mindfulness breath training on depression improvement. Mindfulness training: 1. Mindful breath; 2. Mindful raisins; 3. Mindful eating. |
| **The third and fourth sessions (body mindfulness)** | Participants were guided to experience their own body feeling changes in the practice of mindfulness walking and mindfulness yoga, which made them further understand the relationship between their bodies and symptoms. Mindfulness training: 1. Mindful breath; 2. Body scan. |
| **The fifth and sixth sessions  (emotional mindfulness)** | Participants were trained to understand the relationship between their thoughts, emotional responses, and physical reactions. Moreover, they were guided to explore their automatic thinking patterns and learn to observe the changes in emotions without immersing in the emotions. Mindfulness training: 1. Mindful breath; 2. Emotional meditation. |
| **The seventh and eighth sessions (cognitive mindfulness)** | In these sessions, therapists help participants to perceive the relation between the outside world and their self-feelings, and to identify their own habitual emotional response mode, in order to change their negative thought patterns. Participants were guided to accept their own thoughts and emotions, and integrate the experience learned in the treatment into their daily life. Mindfulness training: 1. Mindful breath; 2. Three minutes of breathing space. |
| **Contents of MBCT** | |
| **A. Mindful Breath** | Last for 15 to 20 minutes each time, using a stable sitting posture, as far as possible not to rely on the back of the chair, back straight, feet on the floor, close eyes, feel the inhalation and exhalation of the lower abdomen bulge and fall, pay attention to the change in breath and the change in inhalation and exhalation, let the breath natural, do not need to control breath, bring the attention back to breath whenever you notice your mind wandering. |
| **B. Body scan** | Last for 30 to 45 minutes each time, relax to sit or lie down, close eyes and follow instructions to pay attention to every part of the body in turn, to perceive the senses of the body at the moment. |
| **C. Mindful yoga** | Last for 10 to 20 minutes each time, integrate the attitude of mindfulness with the simple yoga posture, and focus attention on the body parts of the posture changes and inner feelings at the moment. |
| **D. Mindful walking** | Last for 10 to 20 minutes at a time, walk naturally, keep body relaxed while practicing, and maintain a slow and comfortable pace. During the entire walk, first, perceive the standing posture, then lift the heel of one foot as inhaling, then lower the heel as exhaling, and then lift and lower the other foot as walking, keeping your feet firmly on the ground. Repeat the above steps with both feet in turn. |
| **E. Three-minute breath space** | This part consists of three different modules: First, be aware of body feelings, emotions and thoughts currently; Second, pay attention to breathing, and feel the change in inhalation and exhalation in the present moment. Third, expand attention from breath to the whole body and surroundings. |
| Abbreviation: LLD = late-life depression; MBCT = mindfulness-based cognitive therapy. | |

| **Supplementary Table 2.** **Demographic and clinical characteristics of the MBCT and TAU groups in the MRI analysis.** | | | | |
| --- | --- | --- | --- | --- |
| **Participants** | **MBCT**  **(n = 20)** | **TAU**  **(n = 13)** | **t/χ2** | ***p*** |
|  | **Mean ± SD / n (%)** | **Mean ± SD / n (%)** |  |  |
| **Age (years)** | 66.85 ± 4.71 | 67.18 ± 6.88 | -0.16 | 0.872 |
| **Gender (male)** | 5 (25.00%) | 4 (30.80%) | 0.00 | 1.000 |
| **Education (years)** | 13.55 ± 2.67 | 13.00 ± 3.63 | 0.50 | 0.619 |
| **BMI (kg/m^2^)** | 22.59 ± 2.63 | 23.90 ± 1.44 | -1.63 | 0.113 |
| **Married** | 18 (90.00%) | 12 (92.31%) | -^a^ | 1.000 |
| **Duration of Illness (months)** | 38.45 ± 32.31 | 55.54 ± 36.17 | -1.42 | 0.166 |
| **Age of onset (years)** | 62.90 ± 5.13 | 61.85 ± 6.97 | 0.50 | 0.620 |
| **Comorbidity** |  |  |  |  |
| **Hypertension** | 8 (40.00%) | 5 (38.46%) | 0.01 | 0.930 |
| **Diabetes mellitus** | 1 (5.00%) | 1 (7.69%) | -^a^ | 1.000 |
| **Type of antidepressant** |  |  |  |  |
| **SSRI** | 15 (75.00%) | 10 (76.92%) | 0.00 | 1.000 |
| **SNRI** | 5 (25.00%) | 3 (23.08%) | 0.00 | 1.000 |
| Abbreviation: BMI = body mass index; PA = positive affect; NA = negative affect; MBCT = mindfulness-based cognitive therapy; TAU = treat as usual; SSRI = selective serotonin reuptake inhibitor; SNRI = selective noradrenalin reuptake inhibitors. | | | | |


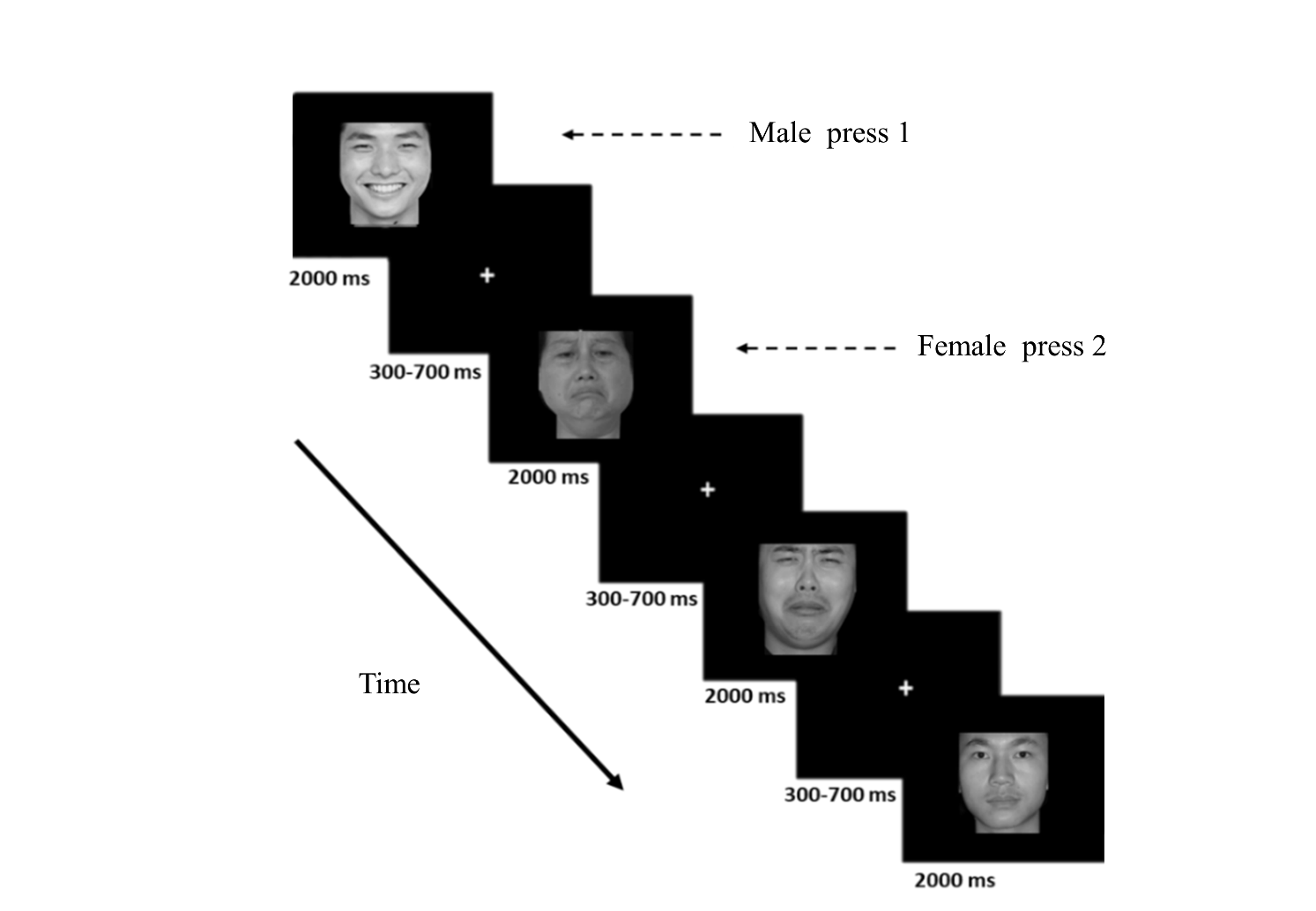


**Supplementary Figure 1. The facial expression recognition task.**
